# *Toxoplasma gondii*, Brazil

**DOI:** 10.3201/eid1303.060599

**Published:** 2007-03

**Authors:** Jorge Gomez-Marin

**Affiliations:** * Universidad del Quindio, Armenia, Quindio, Colombia

**Keywords:** Toxoplasma, risk factors, water transmission, letter

## To the Editor

Recently, Jones et al. reported that past pregnancies increased risk for recent *Toxoplasma gondii* infection in Brazil (*1*). They did not, however, control for age. Previous seroepidemiologic studies have shown that age is a main confounding variable in analysis of risk factors for toxoplasmosis (*2*). Age can explain why mothers with more children are at higher risk for toxoplasmosis; the longer persons live in areas with high toxoplasmosis prevalence, the higher their risk for infection.

Also not explored were drinking water–related factors. Our recent study of pregnant women in Quindio, Colombia, found factors that explained attributable risk percent for infection to be eating rare meat (0.26%) and having contact with a cat <6 months of age (0.19%) (*3*). Drinking bottled water was more significantly protective for the group that did not consume undercooked or raw meat (odds ratio 0.06, 95% confidence interval 0.006–0.560, p = 0.008). We think that drinking water–related factors could explain up to 50% of toxoplasmosis infections in our region.
