# Supplementary material for: Toxoplasma gondii, Brazil
Source: Emerg Infect Dis. 2007 Mar;13(3):512–3. doi: 10.3201/eid1303.060599 (PMC2725897; doi:10.3201/eid1303.060599)
Supplement: Related texts

# Reply: In Response

**Authors:** Jeffrey L. Jones, Cristina Muccioli, Rubens Belfort, Gary N. Holland, Jacquelin M. Roberts, Claudio Silveira

**Affiliations:** * Centers for Disease Control and Prevention, Atlanta, Georgia, USA; † Federal University of São Paulo, São Paulo, Brazil; ‡ University of California, Los Angeles, California, USA; § Clinica Silveira, Erechim, Rio Grande do Sul, Brazil

We thank Dr Gomez-Marin for his letter regarding our article on recently acquired *Toxoplasma gondii* infection in Brazil (*1*). Dr Gomez-Marin states that perhaps age could account for our finding that having had children was a risk factor for recent *T. gondii* infection among women. Studies have shown that age is a risk factor for prevalent *T. gondii* infection; i.e., infection prevalence increases with age (*2*). However, age is not necessarily a risk factor for recent (incident) infection.

Our study of risk factors for *T. gondii* infection was a case-control design to evaluate recent infection, not a cross-sectional study of *T. gondii* infection prevalence in a population. In our study, case-patients with recent infection were similar in age to *T. gondii*–negative control-patients, although among women the mean age of case-patients (33 years) differed slightly from that of control-patients (29 years) (p = 0.03, *t*-test). In addition, multivariate analysis comparing the case-patients with control-patients showed that age was not a significant factor. However, when we kept age in the multivariate model for women (p = 0.87 for age in the model), the odds ratio for having had children changed little, from 14.94 (95% confidence interval [CI] 3.68–60.73) to 14.01 (95% CI 2.88–68.08). Therefore, we do think that, in this study population, having had children is a risk factor for *T. gondii* infection among women.

Dr Gomez-Marin also states that we did not evaluate drinking water–related factors. However, in our methods section (*1*), we indicated that our questionnaire asked about a comprehensive set of risk factors related to drinking water. Specifically, the questionnaire asked about the types of water (city, private well, and others, including bottled water); chlorination; filtering of water; and ingestion of water from streams, lakes, rivers, ponds, or other sources. Although we evaluated numerous water-related factors, we did not find them to be significant in this study, which applies to 1 area of Brazil. In other areas of Brazil, however, studies in which 1 of our authors (J.L.J.) has been involved have found water to be a risk factor or a source of infection (*2*,*3*).

## References

[1] Jones JL, Muccioli C, Belfort R, Holland GN, Roberts JM, Silveira C. Recently acquired *Toxoplasma gondii* infection, Brazil. Emerg Infect Dis. 2006;12:582–7.1670480510.3201/eid1204.051081PMC3294697

[2] Bahia-Oliveira LM, Jones JL, Azevedo-Silva J, Alves CF, Orefice F, Addiss DG. Highly endemic, waterborne toxoplasmosis in north Rio de Janeiro state, Brazil. Emerg Infect Dis. 2003;9:55–62.1253328210.3201/eid0901.020160PMC2873742

[3] de Moura L, Bahia Oliveira L, Wada MY, Jones JL, Tuboi SH, Carmo EH, Waterborne toxoplasmosis, Brazil, from field to gene. Emerg Infect Dis. 2006;12:326–9.1649476510.3201/eid1202.041115PMC3373086
